# Cancer incidence and adverse pregnancy outcome in registered nurses potentially exposed to antineoplastic drugs

**DOI:** 10.1186/1472-6955-9-15

**Published:** 2010-09-16

**Authors:** Pamela A Ratner, John J Spinelli, Kris Beking, Maria Lorenzi, Yat Chow, Kay Teschke, Nhu D Le, Richard P Gallagher, Helen Dimich-Ward

**Affiliations:** 1 School of Nursing, University of British Columbia, 302-6190 Agronomy Road, Vancouver, V6T 1Z3, Canada; 2 BC Cancer Agency Research Centre, 675 West 10th Avenue, Vancouver, V5Z 1L3, Canada; 3 Department of Medicine, University of British Columbia, 890 West 10th Avenue, Vancouver, BC, V5Z 1M9, Canada; 4 School of Environmental Health, University of British Columbia, 2206 East Mall, Vancouver, V6T 1Z3, Canada; 5 School of Population and Public Health, University of British Columbia, 5804 Fairview Avenue, Vancouver, V6T 1Z3, Canada

## Abstract

**Background:**

To determine the relationships of potential occupational exposure to antineoplastic drugs with cancer incidence and adverse pregnancy outcomes in a historical prospective cohort study of female registered nurses (RNs) from British Columbia, Canada (BC).

**Methods:**

Female RNs registered with a professional regulatory body for at least one year between 1974 and 2000 formed the cohort (n = 56,213). The identifier file was linked to Canadian cancer registries. An RN offspring cohort from 1986 was created by linkages with the BC Birth and Health Status Registries. Exposure was assessed by work history in oncology or cancer agencies (method 1) and by estimating weighted duration of exposure developed from a survey of pharmacists and nursing unit administrators of all provincial hospitals and treatment centers and the work history of the nurses (method 2). Relative risks (RR) were calculated using Poisson regression for cancer incidence and odds ratios (OR) were calculated for congenital anomaly, stillbirth, low birth weight, and prematurity incidence, with 95% confidence intervals.

**Results:**

In comparison with other female RNs, method 1 revealed that RNs who ever worked in a cancer center or in an oncology nursing unit had an increased risk of breast cancer (RR = 1.83; 95% CI = 1.03 - 3.23, 12 cases) and their offspring were at risk for congenital anomalies of the eye (OR = 3.46, 95% CI = 1.08 - 11.14, 3 cases). Method 2 revealed that RNs classified as having the highest weighted durations of exposure to antineoplastic drugs had an excess risk of cancer of the rectum (RR = 1.87, 95% CI = 1.07 - 3.29, 14 cases). No statistically significant increased risks of leukemia, other cancers, stillbirth, low birth weight, prematurity, or other congenital anomalies in the RNs' offspring were noted.

**Conclusions:**

Female RNs having had potential exposure to antineoplastic drugs were not found to have an excess risk of leukemia, stillbirth, or congenital anomalies in their offspring, with the exception of congenital anomalies of the eye, based on only three cases; however, elevated risks of breast and rectal cancer were observed.

## Background

The nursing profession is known to involve occupational exposures that may have adverse health effects. Lie and Kjæheim's [1] review of 19 published epidemiological studies of nurses, conducted between 1983 and 2001, concluded that RNs may be at increased risk for breast cancer and leukemia related to their work. Some studies have shown that the offspring of nurses who continue to work during pregnancy are at increased risk of prenatal development of congenital anomalies [2,3].

More specifically, there has been concern regarding the potential for carcinogenic and teratogenic effects related to antineoplastic drugs exposure. Antineoplastic drugs have been used in the treatment of malignant diseases for more than 50 years. There are almost 100 antineoplastic drugs currently in use, many of which are mutagenic and either known or probable human carcinogens [4]. Second malignancies, including leukemia and bladder cancer, have been reported in patients who have previously received antineoplastic drugs [4]. Nurses employed in oncology treatment programs may be occupationally exposed to antineoplastic drugs through inhalation of aerosolized drug products and by direct skin contact of residues on drug vials, contaminated intravenous tubing, drug spillage, and patients' excreta (urine, feces, blood, or vomitus), or orally through hand-to-mouth contact [5]. Although the intensities of exposure are much less than those of patients receiving treatment, occupational exposures typically are of longer cumulative duration. Despite the introduction of safety guidelines and protective measures, health-care workers can still be exposed to these toxic drugs, as demonstrated by detectable levels of biomarkers found in the urine of nurses and other health professionals, and DNA damage or chromosomal aberrations observed in their lymphocytes and exfoliated buccal cells [6-9]. Mutagenic effects of antineoplastic drugs provide an explanatory mechanism for elevated risks of cancer and adverse reproductive outcomes. Antimetabolites, a commonly used class of antineoplastic drugs, may also affect reproductive outcomes through folate antagonist activity [10,11].

Several epidemiological studies have been conducted of the health risks of nurses, in general, as a result of antineoplastic drug exposure. Increased risks of leukemia and breast cancer were suggested in two studies of nurses who handled antineoplastic drugs [12,13]. A 1985 study showed an increased risk of congenital anomalies in the offspring of RNs exposed to antineoplastic drugs [14]. Fransman et al. [15], however, reported that RNs exposed to antineoplastic drugs, relative to other nurses, had difficulty conceiving, and were at greater risk of premature delivery and low birth weight infants, although not at greater risk of spontaneous abortion, stillbirth, or congenital anomalies.

We had the opportunity to examine a very large cohort of registered nurses (RNs) and their offspring in relation to this occupational exposure. The objectives of our study were to determine whether a cohort of female RNs employed since 1974 by oncology/cancer agencies or by any nursing departments determined to have possible or probable exposure to antineoplastic drugs, in the province of BC, had increased risks of cancer (breast cancer or leukemia, in particular) or adverse pregnancy outcomes, including stillbirth, congenital anomalies, low birth weight, and prematurity.

## Methods

### Study Population

The cohort, described previously [16], consisted of 56,213 females registered with their professional regulatory body for at least one year between 1974 and 2000 in the province of BC. The cohort file was first linked with the Canadian Cancer Registry. Observations were censored for RNs lost to follow-up (i.e., no longer registered and known to have left Canada before the end of the study period). Otherwise, they were assumed to be cancer-free at the end of 1999, unless there was a cancer diagnosis reported in the cancer registry. The exposure period was lagged 10 years prior to the incident cancer cases to allow for a minimum latency period of 10 years. The cancer cases were categorized by site according to the *World Health Organization's *9^th ^edition of the International Classification of Disease (ICD-9).

The cohort file was linked to the live and stillbirth records of the BC Vital Statistics Agency to create an RN offspring cohort of 22,491 live and 115 stillbirths. Records of birth weight and gestational age at birth enabled the identification of offspring with low birth weight and those born prematurely. Stillbirth was defined as the delivery of a dead fetus following 20 or more weeks of gestation. Low birth weight included offspring weighing less than 2500 grams and premature birth included offspring born at a gestational age of less than 37 weeks. The offspring cohort was linked to the BC Health Status Registry (HSR) to obtain information about the presence of congenital anomalies diagnosed any year (up to 20 years) after birth and, if present, their type, as categorized according to the ICD-9 classification system. The HSR was established in 1952 with a mandate to ascertain, record, and classify "handicapping" conditions, congenital anomalies, and genetic defects in the population. Because recording practices were less consistent in the earlier periods of surveillance, BC Vital Statistics limited access to HSR data to the interval, 1986 to 2000, to improve upon the consistency in data quality. Cases were limited to live, singleton offspring to prevent potential confounding from the adverse effects associated with stillbirth and multiple-birth pregnancies.

The study was approved by the University of British Columbia's Clinical Research Ethics Board.

### Exposure Assessment

For all cohort members, the work history file derived from the annual registration renewal records contained the following information for each year the RNs were registered to practice in the province: name of employer, type of employer, employment position, level of nursing education attained, primary area of responsibility, full or part-time status, and number of hours worked.

Exposure to antineoplastic drugs was assessed by two methods. The first method (method 1) calculated the number of years the RN had been employed in the field of oncology or by the provincial cancer agency, as reported in the annual registration renewal records. For these RNs, the BC Cancer Agency (the organization that coordinates all provincial cancer centers) was recorded as the place of employment for the entire study period, or the nurses reported that they had been employed in oncology by another hospital. Oncology was added to the registry as a field of employment in 1996. The BC Cancer Agency was listed as a place of employment for the full study period. The majority of nurses in oncology were employed outside of the BC Cancer Agency (71.0% for the years 1996-2000), typically in a general hospital setting. Before 1996, nurses employed in oncology outside of the BC Cancer Agency could not be identified. For the calculation of total years exposed, full-time employment was assigned a weight of 1.0 and part-time employment was assigned a weight of 0.5.

**Figure 1:**
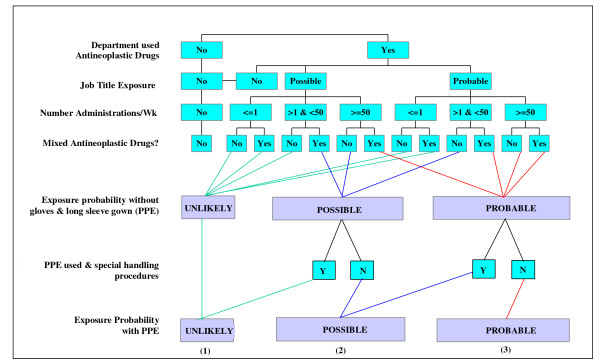
**Algorithm of probability of antineoplastic drug exposure**. Algorithm used for individual assessment of probability of exposure, based on survey questions administered to head nurses of each department included in the study. Based on responses displayed in the boxes to each of the listed, ordered questions, nurses employed by a given department were allocated in a deterministic manner to either having unlikely, possible, or probable exposure to antineoplastic drugs.

A second method of ascertaining exposure to antineoplastic drugs (method 2) was based on telephone interviews with pharmacists and with senior nurses in nursing departments from all 94 general hospitals (357 departments) and 19 diagnostic and treatment centers in BC. Pharmacists were asked about the periods of time antineoplastic drugs were used at the facility and about the frequency of use of up to 74 individual antineoplastic drugs. Senior nurses were asked whether any nurses in their department had administered or mixed antineoplastic drugs or cared for patients who received the drugs. Positive responses were followed by further questions about the probability of exposure (i.e., no exposure, possible exposure, or probable exposure) by nursing position (i.e., supervisor/coordinator, clinical nurse specialist, head nurse/unit manager, charge nurse, staff nurse) and year. For all positions and years with possible and probable exposures, these senior nurses were asked whether specific personal protective equipment was used, and whether waste disposal, spill, and patient care guidelines were in place and followed. Finally, if nurses in the department were involved in the mixing of antineoplastic drugs, the location of the procedure was queried (i.e., bio-safety cabinet, laminar flow hood, desk/nursing station, or medication room). Exposure to antineoplastic drugs was then classified as no, unlikely, possible, or probable using an algorithm created by the study hygiene team and completed by the occupational hygienist (see Figure 1). The algorithm used the available published evidence about factors influencing exposures, and was applied to the following departmental survey data elements for each nursing position and year: probability that the job involved antineoplastic drug administration; the number of times per week the drugs were administered; whether the mixing of antineoplastic drugs was performed; whether gloves and long-sleeved gowns were used; and whether special handling procedures were followed for waste disposal, patient handling, and spill clean-up.

A weighted exposure duration was calculated for each nurse's working history during the cohort period. To acknowledge the skewed distributions of exposures of nurses to antineoplastic agents [17], and to ensure that only those with reasonable possibilities of exposure were attributed exposure-time, a multiplicative weighting (0.00, 0.0625, 0.25, and 1.00) was assigned to the exposure probabilities from the algorithm (none, unlikely, possible, and probable exposure, respectively). In addition, part-time employment was assigned a weight of 0.5 and full-time employment a weight of 1.0. Weighted exposure durations were cumulated over the person's full job history, as follows:

$$\sum_{j=1}^{n}P_{j}\times H_{j}$$

where *j *is the specific job in a specific department for a specific employer for a specific year, *P_j _*is the weight for the assigned probability of exposure for that job/department/employer/year and *H_j _*is the weight for full-time and part-time employment. A weighted duration of exposure of 15 days is equivalent to full-time work for one year (240 days) at a job considered unlikely to be exposed; a weighted duration of exposure of 60 days is equivalent to full-time work for one year at a job considered possibly exposed.

Exposure assessment for the birth outcomes analysis was determined by the above format for two measures: (a) estimated exposure during the first trimester of pregnancy and (b) cumulative exposure over the ten years preceding the date of birth.

### Statistical Analysis

Poisson regression analysis was performed on the cancer incidence of the RN cohort, using the R statistical software package [18]. Adjustment was made for calendar year and the age of the RN, and a lag of 10 years was applied. Based on the distribution of cumulative work years, the years of work in oncology for method 1 were categorized into 3 groups: (a) none, (b) < 5 years, and (c) ≥ 5 years. Weighted duration of exposure to antineoplastic drugs for method 2 was also categorized into 3 groups: (a) < 15 days, (b) ≥ 15 to 60 days, and (c) > 60+ days.

Logistic regression analysis was performed on the reproductive outcomes of the offspring cohort, using SPSS 16 (SPSS Inc., Chicago, IL). Adjustments were made for the age of the mother at birth, year of birth, and the sex of the child. Because of the relatively small number of births with adverse reproductive outcomes, the years of work in oncology or a cancer center (method 1) were categorized into 2 groups ("none" or 1+ years ("ever")), and exposure to antineoplastic drugs (method 2) was collapsed into two groups (< 15 days and ≥ 15 days).

For all analyses, two-sided testing with a 5% significance level was used, corresponding to a 95% confidence interval.

## Results

Of the 56,213 female RNs who had a work history between 1974 and 2000, 905 (1.6%) RNs were identified as ever having been exposed to antineoplastic drugs according to method 1, based on their employment in oncology nursing units or at a cancer center, and 7,635 (13.6%) of the 56,213 RNs were assigned at least 15 days weighted duration of exposure to antineoplastic drugs according to method 2, based on information acquired through the agency-based survey.

During 1986-2000, 12,741 RNs gave birth to 22,491 live and singleton offspring, which defined the offspring cohort (see Table 1). Sixty nine RNs were identified as having worked in oncology for the time it was available as a field code (1996-2000), of which 20 (29.0%) were also identified as having worked in a cancer center during pregnancy. A total of 141 were identified as having worked in a cancer center during pregnancy.

**Table 1:** Descriptive characteristics of the RN offspring cohort, 1986-2000.

	N	%
Total births	23,222	
Singletons	22,613	97.4%
Stillbirths^1^	147	0.6%
Twins	581	2.5%
Triplets	28	0.1%
		
Live singletons	22,491	
Only child	9,163	40.7%
2 children	8,454	37.6%
≥ 3 children	4,874	21.7%
Males	10,961	48.7%
Females	11,530	51.3%
		
RN mothers	12,741	
		
Mother's age at birth		
< 30 years	7,943	35.3%
30 - 34 years	9,371	41.7%
≥ 35 years	5,177	23.0%

^1^Value includes twins and triplets, and cases with missing or incomplete congenital anomaly diagnosis data.

As shown in Table 2, having ever worked in oncology or a cancer center (method 1) was associated with a significantly elevated relative risk of breast cancer (RR = 1.83, 95% CI = 1.03 - 3.23, 12 cases). The risks for all cancers (RR = 1.28, 95% CI = 0.83 - 1.79, 21 cases), or for cancers specifically of the uterus (RR = 2.58, 95% CI = 0.96 - 6.94, 4 cases), were elevated, but were not statistically significant. Cases of other cancers had frequencies below 3 and no cases of leukemia were observed among those having worked in oncology or a cancer center.

**Table 2:** Relative risk of selected cancer incidence of female registered nurses according to cumulative years worked in oncology or a cancer center (method 1), 1996-2000 (N = 56,213)^1,2,3,4^

Site (ICD-9 code)	Employment Duration	Observed	RR	CI (95%)	p-value^5^
All cancers except non-melanoma skin	never	3078	1.00		0.135
	< 5 years	14	1.10	0.65-1.87	
	> 5 years	7	1.89	0.90-3.97	
	ever	21	1.28	0.83-1.79	
					
Breast (174)	never	1274	1.00		0.379
	< 5 years	9	1.75	0.91-3.38	
	> 5 years	3	2.10	0.67-6.53	
	ever	12	1.83	1.03-3.23	
					
Uterus (179-182)	never	302	1.00		0.068
	< 5 years	3	2.50	0.80-7.82	
	> 5 years	1	2.85	0.40-20.38	
	ever	4	2.58	0.96-6.94	

^1^Based on Poisson regression using a 10-year lag.

^2^Cancers with an incidence of less than 3 cases were excluded.

^3^N for ever worked in oncology or cancer center = 905.

^4^Adjusted for age and calendar year.

^5^Significance of the trend, derived from chi-square analysis.

Table 3 shows the relative risk of cancer by probability of exposure to antineoplastic drugs (method 2). For those in the top two exposure categories, the risk of rectal cancer was significantly elevated, based on 14 cases (RR = 1.87, 95% CI = 1.07 - 3.29), and the risk of breast cancer was elevated, but was not statistically significant (RR = 1.12, 95% CI = 0.89 - 1.39, 87 cases). The risks for all cancers (RR = 1.08, 95% CI = 0.93 - 1.25, 194 cases), and for each other type of cancer, were not statistically significantly elevated. Three cases of leukemia were observed among those in the top exposure categories.

**Table 3:** Relative risk of selected cancer incidence for female registered nurses according to weighted duration of exposure to antineoplastic agents (method 2), 1974-2000 (N = 56,213)^1,2,3,4^

Site (ICD-9 code)	Weighted duration of exposure^5^	Observed	RR	CI (95%)	p-value^6^
All cancers except	< 15 days	2905	1.00		0.452
non-melanoma skin	15 to < 60 days	150	1.11	0.94-1.30	
	≥ 60 days	44	1.00	0.74-1.35	
	top 2 categories combined	194	1.08	0.93-1.25	
					
Colon (153,159)	< 15 days	225	1.00		0.710
	15 to < 60 days	11	1.16	0.63-2.13	
	≥ 60 days	< 3			
	top 2 categories combined	13	0.99	0.56-1.74	
					
Rectum (154)	< 15 days	116	1.00		0.037
	15 to < 60 days	10	1.85	0.96-3.56	
	≥ 60 days	4	1.93	0.71-5.25	
	top 2 categories combined	14	1.87	1.07-3.29	
					
Lung (162)	< 15 days	202	1.00		0.867
	15 to < 60 days	11	1.24	0.67-2.28	
	≥ 60 days	3	0.83	0.27-2.61	
	top 2 categories combined	14	1.22	0.65-1.94	
					
Melanoma (172)	< 15 days	191	1.00		0.400
	15 to < 60 days	12	1.28	0.71-2.30	
	≥ 60 days	3	1.28	0.41-4.02	
	top 2 categories combined	15	1.28	0.75-2.18	
					
Breast (174)	< 15 days	1199	1.00		0.267
	15 to < 60 days	64	1.08	0.84-1.39	
	≥ 60 days	23	1.23	0.82-1.87	
	top 2 categories combined	87	1.12	0.89-1.39	
					
Uterus (179-182)	< 15 days	290	1.00		0.696
	15 to < 60 days	9	0.72	0.37-1.40	
	≥ 60 days	7	1.66	0.78-3.53	
	top 2 categories combined	16	0.95	0.57-1.59	
					
Ovary (183)	< 15 days	144	1.00		0.209
	15 to < 60 days	6	0.90	0.40-2.06	
	≥ 60 days	< 3			
	top 2 categories combined	6	0.68	0.30-1.55	
					
Bladder - including in-situ (188)	< 15 days	78	1.00		0.394
	15 to < 60 days	6	2.00	0.86-4.65	
	≥ 60 days	< 3			
	top 2 categories combined	7	1.67	0.76-3.67	
					
Brain (191-192)	< 15 days	43	1.00		0.292
	15 to < 60 days	3	1.76	0.54-5.80	
	≥ 60 days	< 3	1.88	0.26-13.87	
	top 2 categories combined	4	1.79	0.63-5.10	
					
Thyroid (193)	< 15 days	82	1.00		0.408
	15 to < 60 days	4	0.93	0.34-2.56	
	≥ 60 days				
		< 3			
	top 2 categories combined	4	0.74	0.27-2.05	
Ill-defined (195)	< 15 days	81	1.00		0.181
	15 to < 60 days	5	1.33	0.54-3.31	
	≥ 60 days	3	2.09	0.66-6.65	
	top 2 categories combined	8	1.54	0.74-3.21	
					
Lymphatic & Hematopoietic	< 15 days	219	1.00		0.482
(200-208)	15 to < 60 days	9	0.93	0.47-1.82	
	≥ 60 days	< 3			
	top 2 categories combined	11	0.84	0.46-1.55	
Non-Hodgkin Lymphoma	< 15 days	121	1.00		0.643
(200, 202)	15 to < 60 days	6	1.06	0.47-2.43	
	≥ 60 days	< 3	0.49	0.07-3.52	
	top 2 categories combined	7	0.91	0.42-1.96	
Leukemia (204-208)	< 15 days	59	1.00		0.930
	15 to < 60 days	< 3			
	≥ 60 days	< 3			
	top 2 categories combined	3	0.89	0.27 - 2.88	

^1 ^Based on Poisson regression using a 10-year lag.

^2 ^Cancer with an incidence of less than 3 were excluded.

^3 ^N for top 2 categories of antineoplastic drug exposure = 7,635.

^4 ^Adjusted for age and calendar year.

^5 ^A weighted duration of exposure of 15 days is equivalent to full-time work for one year (240 days) at a job considered unlikely to be exposed; a weighted duration of exposure of 60 days is equivalent to full-time work for one year at a job considered possibly exposed.

^6 ^Significance of the trend, derived from chi-square analysis.

Of 22,491 live births of the offspring cohort, 1,391 (6.2%) were diagnosed with congenital anomalies. One hundred and seventy (0.8%) mothers (n = 12,741) were identified as having worked in oncology nursing units or in a cancer center between 1986 and 2000 during pregnancy. A total of 2,650 (11.8%) of the mothers in the cohort were assigned as having had at least 15 days weighted duration of exposure to antineoplastic drugs in the 10 years preceding the pregnancy.

Among the offspring of mothers who had ever worked in oncology nursing units or a cancer center during their pregnancy (method 1), the risk of all congenital anomalies or congenital anomalies of the circulatory or musculoskeletal systems were elevated, but not statistically significantly; the risk of anomalies of the eye was statistically significantly elevated at 3.46 (95% CI = 1.08 - 11.14), but based on only 3 cases (see Table 4). As shown in Table 5, there were no statistically significant elevated risks of congenital anomalies among the offspring of mothers with exposure to antineoplastic drugs during the first trimester of pregnancy or over the 10 years preceding pregnancy (method 2). However, the risk of cleft palate or lip, for the 10-year exposure period, was notably high, at 1.84 (95% CI = 0.75 - 4.49, 6 cases). Stillbirths were an infrequent outcome for the offspring cohort, with only 115 cases. There was no increased risk of stillbirth related to antineoplastic drug exposure during the first trimester of pregnancy (OR = 0.67, 95% CI = 0.21 - 2.13, 3 cases) or during the 10 years preceding pregnancy (OR = 0.63, 95% CI = 0.31 - 1.30, 8 cases). There were no cases among those who had worked in oncology nursing units or for a cancer center during pregnancy.

**Table 4:** Risk of congenital anomalies among the offspring of RNs employed in oncology nursing units or a cancer center during pregnancy (method 1), 1996-2000 (N = 22,491)^1,2^

Congenital anomaly category (ICD9 code)	Employed	Cases	OR^3^	CI (95%)
All congenital anomalies (740-759)	never	1024	1.00	
	ever	17	1.42	0.86-2.36
				
Eye (743)	never	69	1.00	
	ever	3	3.46	1.08-11.14
				
Circulatory system (747)	none	94	1.00	
	ever	3	2.68	0.84-8.59
				
Musculoskeletal system	never	335	1.00	
(754-756)	ever	5	1. 21	0.49-2.96

^1^Congenital anomaly types with an incidence of less than 3 cases were excluded.

^2^N for ever employed in oncology or cancer center = 190.

^3^Adjusted for sex, year of birth, and age of mother.

**Table 5:** Risk of congenital anomalies among the offspring of RNs according to weighted duration of exposure to antineoplastic drugs (method 2), 1986-2000 (N = 22,491)^1,2^

Congenital anomaly category (ICD9 code)	Weighted duration of exposure^3^	Exposure during first trimester of pregnancy			Exposure during 10 years preceding pregnancy		
		cases	OR^4^	CI (95%)	cases	OR^4^	CI (95%)
All congenital anomalies	< 15 days	1,328	1.00		1229	1.00	
(740-759)	≥ 15 days	63	0.93	0.72-1.21	162	0.98	0.82-1.16
							
Nervous system	< 15 days	51	1.00		47	1.00	
(740-742)	≥ 15 days	< 3			6	0.96	0.41-2.24
							
Eye	< 15 days	89	1.00		81	1.00	
(743)	≥ 15 days	6	1.31	0.57-3.00	14	1.26	0.71-2.22
							
Ear, face, neck	< 15 days	85	1.00		82	1.00	
(744)	≥ 15 days	4	0.96	0.35-2.64	7	0.64	0.30-1.39
							
Heart	< 15 days	137	1.00		127	1.00	
(745-746)	≥ 15 days	10	1.44	0.75-2.74	20	1.15	0.72-1.84
							
Other circulatory system	< 15 days	127	1.00		115	1.00	
(747)	≥ 15 days	8	1.26	0.61-2.58	20	1.28	0.79-2.06
							
Cleft palate/lip	< 15 days	29	1.00		25	1.00	
(749)	≥ 15 days	< 3			6	1.84	0.75-4.49
							
Upper alimentary tract	< 15 days	83	1.00		81	1.00	
(750)	≥ 15 days	4	0.93	0.34-2.55	6	0.55	0.24-1.25
							
Other digestive system	< 15 days	35	1.00		32		
(751)	≥ 15 days	< 3			5	1.17	0.46-3.01
							
Genital organs	< 15 days	213	1.00		192	1.00	
(752)	≥ 15 days	6	0.55	0.24-1.25	27	1.06	0.70-1.59
							
Urinary system	< 15 days	101	1.00		93	1.00	
(753)	≥ 15 days	5	0.97	0.39-2.38	13	1.05	0.59-1.88
							
Musculoskeletal system	< 15 days	443	1.00		419	1.00	
(754-756)	≥ 15 days	18	0.80	0.49-1.28	42	0.74	0.54-1.02
							
Integument	< 15 days	96	1.00		88	1.00	
(228, 757)	≥ 15 days	5	1.01	0.41-2.49	13	1.08	0.60-1.94
							
Chromosomal anomalies	< 15 days	60	1.00		56	1.00	
(758)	≥ 15 days	< 3			4	0.54	0.20-1.49
							
Multiple anomalies	< 15 days	15	1.00		13	1.00	
(759.7-759.8)	≥ 15 days	< 3			3	1.76	0.50-6.19

^1 ^Congenital anomalies with an incidence of less than 3 cases were excluded.

^2 ^N for ≥ 15 days of antineoplastic drug exposure during first trimester of pregnancy = 1,062; during 10 years preceding pregnancy = 2,650.

^3 ^A weighted duration of exposure of 15 days is equivalent to full-time work for one year (240 days) at a job considered unlikely to be exposed; a weighted duration of exposure of 60 days is equivalent to full-time work for one year at a job considered possibly exposed.

^4 ^Adjusted for sex, year of birth, and age of mother.

There were 746 cases of low birth weight and 1,133 cases of prematurity among the offspring cohort. Among those employed in oncology or a cancer center during pregnancy, the risks of low birth weight (OR = 1.41, 95% CI = 0.44 - 4.54, 3 cases) and prematurity (OR = 1.88, 95% CI = 0.80 - 4.41, 6 cases) were both elevated, but were not statistically significant. There were no increased risks of low birth weight or prematurity, respectively, related to antineoplastic drugs exposure during the first trimester of pregnancy (OR = 0.97, 95% CI = 0.43 - 2.20, 6 cases; OR = 1.05, 95% CI = 0.56 - 2.00, 10 cases) or during the 10 years preceding pregnancy (OR = 0.67, 95% CI = 0.32 - 1.42, 7 cases; OR = 0.62, 95% CI = 0.33 - 1.17, 10 cases).

## Discussion

This cohort of RNs who worked in BC between 1974 and 2000 showed a slightly increased but non-significant risk of all cancers if they had potential exposure to antineoplastic drugs. The risk of breast cancer was significantly elevated among those who had ever worked in oncology nursing units or for a cancer center, which showed a possible trend of increasing incidence with increasing years of work. However, this increase was elevated but not statistically significant in RNs classified as potentially exposed to antineoplastic drugs, based on a survey-based exposure assessment protocol. An increased risk of cancer of the rectum was observed in RNs determined to have had a probability of exposure to antineoplastic drugs. No increased risk of any other cancer type, including leukemia, was observed using either exposure assessment method.

It has been well established that nurses in oncology who handle antineoplastic drugs have increased frequencies of biomarkers of exposure, including chromosomal aberrations and sister chromatid exchanges [19-22]. However, to date, very few relevant epidemiological studies have estimated RNs' cancer risk related to exposure to antineoplastic drugs. A linkage study of Danish nurses who worked in oncology departments, preparing and administering antineoplastic drugs, reported a statistically significantly increased relative risk (RR = 10.65) for one site, leukemia, based on 2 cases [12]. A nested case-control study of 59 breast cancer cases from an Icelandic cohort study of female nurses found that the risk estimates were highest (although not statistically significant and based on 7 cases) among those who had ever handled cytotoxic drugs (OR = 1.65, 95% CI = 0.53 - 5.17), after adjustment for year of birth, breast cancer in a first-degree relative, marital status, and nulliparity [13]. Our finding of a significantly increased risk of breast cancer among RNs who had ever worked in oncology or a cancer center provides some additional evidence of this association and supports the relevance of further investigating occupational risk factors for nurses. In future research about nursing and cancer incidence, consideration should also be given to the possible influence of mammography screening rates. It is possible that oncology nurses, who directly care for women with breast cancer, are predisposed to mammography screening participation and thus are subject to an over-detection bias. It is recognized that some breast cancers, if undetected and thus not treated, would not progress and that some women are "over-diagnosed" [23,24].

The mutagenic effects of antineoplastic drugs may increase the risk of congenital anomalies and stillbirth among exposed nurses. Of three case-control studies, one showed an odds ratio of 4.7 (95% CI = 1.2 - 18.1) for congenital anomalies among those exposed to antineoplastic drugs at least once a week [14], one showed a relative risk of 1.7 (95% CI = 1.0 - 2.8) for spontaneous abortions among occupationally exposed nurses [25], and a third showed an odds ratio of 2.3 (95% CI = 1.2 - 4.4) for fetal loss among nurses exposed during their first trimester of pregnancy [26]. A cross-sectional study observed 8 congenital anomalies among offspring of 152 physicians and nurses who administered antineoplastic drugs during pregnancy (4.05 expected; *p *= 0.05) [27]. More recent studies have shown few reproductive risks from antineoplastic drug exposure. A questionnaire-based study found log-linear odds ratios of 1.20 (95% CI = 0.98 - 1.47) for stillbirth and 0.97 (95% CI = 0.86 - 1.09) for congenital anomalies among the offspring of nurses exposed to antineoplastic drugs [12]. Two case-control studies found odds ratios of 1.02 (95% CI = 0.47 - 2.06) and 2.2 (95% CI = 0.7 - 7.2) for congenital anomalies among the offspring of nurses who handled antineoplastic drugs, compared with nurses without exposure [12,28]. A recent meta-analysis of four studies of nurses' exposure to antineoplastic drugs estimated an odds ratio for congenital anomaly incidence of 1.64 (95% CI = 0.91 - 2.94) [7].

In our study, the risk of congenital anomalies of the eye was significantly increased in the offspring of RNs who had ever worked in oncology nursing units or for cancer centers during pregnancy (method 1), although this estimate was based on only 3 cases. The risks for other congenital anomalies among this employment group were also notably elevated, but not statistically significantly and were limited by the low frequency of cases. The comparatively higher risk of all congenital anomalies in the employment group may suggest an oncology/cancer agency-specific effect that is perhaps diluted in the broader department-based exposure assessments. The risks of stillbirth, low birth weight, and prematurity were not significantly increased among RNs potentially exposed to antineoplastic drugs, assessed through either their employment in oncology or estimated weighted duration of exposure.

According to the BC Cancer Agency Benefit Drug List, there was an increase in the number of different antineoplastic drugs used, from 66 in 1986 to 88 in 2000. Standardized safety practices for handling antineoplastic drugs and other hazardous materials were established in 1985. However, a recent review of methods used to monitor occupational exposure to antineoplastic drugs concluded that, despite the introduction of safety guidelines and protective measures, health-care workers can still be exposed [8]. Nonetheless, the adoption of these practices may partly account for the predominantly null or non-significant risks observed in this and other recent studies [15,12,28].

Whereas many previous studies were questionnaire-based and were, therefore, susceptible to exposure recall and selection biases, our record linkage used comprehensive recruitment of an employment group based on registry-derived data and allowed for complete ascertainment of outcomes according to standardized medical reporting systems. Furthermore, the historical prospective design of the study meant that exposure variables were assessed independently from health outcomes.

Despite our large sample size, limitations of our study arise from limited statistical power associated with the small numbers of cases and the low prevalence of exposure, possibly leading to missed associations, as well as the testing of multiple hypotheses, such that a statistically significant association may have occurred by chance alone.

As is common in historical cohort studies, we had no information about potential confounding factors related to lifestyle; however, all comparisons were within the nursing profession, a narrow, well educated, socioeconomic stratum. Nurses have been documented to have healthier habits than the population as a whole, but there have been temporal patterns, for example strong declines in smoking rates among US nurses in the last 30 years (33% smokers in 1976 vs. 8.4% in 2002/2003 [29]). There also were temporal patterns in antineoplastic drug use in the British Columbia healthcare agencies during the study period (e.g., hand mixing of the drugs was completed in 45% of the facilities in the 1970 s and 1980 s, but only 8% in the 1990 s and later). If smoking and antineoplastic drug exposures were correlated and both related to the outcomes of interest, there could be uncontrolled confounding in the study results. In a recent study of 1,147 live births among nurses, controlling for parity, smoking, alcohol, coffee, multivitamin, and folic acid intake did not materially change the effect estimates for congenital anomalies [15].

We were not able to adjust for other chemical exposures in the RNs' workplaces or other environments. Unfortunately, there is little if any data available about the presence and usage of such chemicals. We are left to assume that the RNs exposed to antineoplastic drugs were similar to the unexposed RNs with respect to their opportunity to having been exposed to other toxins.

The exposure assessment methods used in the study were crude and may have resulted in misclassification of exposure. For example, in method 1, most nurses working in oncology departments were outside of the cancer centers and were not identifiable in the registry, prior to 1996. This likely led to underestimation of the number of exposed RNs but would have been highly specific. The survey (method 2) identified relevant departments in general hospitals and treatment centers that administered antineoplastic drugs and ascribed individual nurses therein with a probability of exposure. Within these departments, some of the nurses ascribed a probability of exposure may not have been exposed and, within other departments, some of the nurses may have been missed because of poor information recall. This likely resulted in misclassification, which would have biased the effect estimate toward the null. Nonetheless, the method was novel in its attempt to more precisely characterize exposure estimates of a cohort of nurses through the use of relevant questions about employment history that were simple enough to assume sufficient recall for an approximation of exposure; that is, the classification was broadly based.

The premise underlying method 1 was that the oncology departments, as cancer treatment facilities by definition, would have consistently used antineoplastic drugs, whereas other departments (in general hospitals and other agencies) may not have used them, or when they did, perhaps they did so with less regularity. This method more definitively assigned exposure through the use of reliable employment records, limited the exposure group, and introduced some uncertainty about the unexposed group (it may have contained nurses from nursing departments with possible exposure).

Consequently, these limitations of the assessments of exposure may have affected their accuracy and could possibly have resulted in an underestimation of the risks associated with exposure to antineoplastic drugs or have distorted the shape of the dose-response relationship. For instance, the relative risk found for breast cancer using method 2, as opposed to method 1, was elevated, but not statistically significant.

## Conclusions

In summary, RNs potentially exposed to antineoplastic drugs through their employment had an elevated risk of breast and rectal cancer. There was no demonstrated risk of other types of cancer or of adverse reproductive outcomes, apart from an elevated risk for anomalies of the eye based on three cases. Further investigations, including more detailed measurements of exposures and dosages received, may be helpful in evaluating the mechanisms by which occupational exposures to antineoplastic drugs may act as risk factors for cancer and adverse reproductive outcomes.

## Competing interests

The authors declare that they have no competing interests.

## Authors' contributions

HD conceived of the study proposal and participated in the draft construction; KB performed background literature searches; PR, JS, RG, KB, and NL participated in the draft construction; ML, YC, and KB conducted the data analyses. All authors read and approved of the final manuscript.

## Pre-publication history

The pre-publication history for this paper can be accessed here:

http://www.biomedcentral.com/1472-6955/9/15/prepub
